# Patient Satisfaction in the Spanish National Health Service: Partial Least Squares Structural Equation Modeling

**DOI:** 10.3390/ijerph16244886

**Published:** 2019-12-04

**Authors:** María del Carmen Valls Martínez, Alicia Ramírez-Orellana

**Affiliations:** Department of Economics and Business, University of Almería, 04120 Almeria, Spain; aramirez@ual.es

**Keywords:** National Health Service, patient satisfaction, health policy, quality of healthcare, partial least squares structural equation modeling (PLS-SEM)

## Abstract

The aim of this article was to determine which key indicators influence patient satisfaction with the Spanish NHS to provide useful information for policy decision-making. A total of 33 variables for each of the 17 Spanish autonomous communities were collected from the statistical portal of the Spanish Ministry of Health, Social Services, and Equality between 2005 and 2016. A cross-sectional study was applied using Partial Least Squares to a Structural Equation Model (PLS-SEM). The influence of expenditures, resource allocation, and safety were hypothesized about patient satisfaction. Gross Domestic Product (GDP) and life expectancy were used as control variables. Moreover, the influence of resource allocation on use was tested. The model explained 57.1% of patient satisfaction with the Spanish NHS. It was positively influenced mainly by resource allocation and expenditures, followed by safety and life expectancy. Additionally, resources directly influenced the level of use. The number of hospital beds, hemodialysis equipment, rate of adverse drug reactions, and expenditure positively influenced patient satisfaction. In contrast, the number of posts in day hospitals, the hospital infection rate, and the percentage of pharmacy spending negatively influenced patient satisfaction.

## 1. Introduction

An excellent healthcare service contributes to improving the health of population in a specific country by increasing the expectancy of life of its citizens, promoting its social equity, and enhancing the efficiency of its economy [1]. The main aim of National Health Service (NHS) is to achieve a healthier population, that is, to improve citizens’ quality of daily life and health. This implies an essential challenge to the health managers, who have to face an increasingly demanding population and the rising cost of medical technology and resources that are not only limited and scarce, but also reduced as a consequence of the economic crisis. The system is complex and there are many factors to consider, so its management is difficult, and this difficulty is increased by the limited availability of data [2].

Indeed, in recent years, the economic crisis has led governments to reduce investment in public services, including healthcare service, while citizens are increasingly interested in the management and quality of the services they receive, the cost of which is paid through taxes. Therefore, the decrease of the budget in the provision of certain services can affect citizen satisfaction with the NHS [3].

Since the time of Hippocrates, there has been concern regarding the quality of healthcare [4] because quality is vital for patient satisfaction and the success of the health industry [5]. A higher quality is identified by greater patient satisfaction [6,7]. In fact, healthcare is a sector where low quality has serious consequences [8], so customers (patients) are more intolerant of poor service quality than in other sectors [9]. Service quality is an important and increasing issue of research in management, especially health service quality, which is more important than other services [10].

According to Numbeo [11], in mid-2019, the healthcare index for countries ranked from 86.89 in Taiwan to 39.35 in Morocco, out of the 89 countries considered (Table 1). Europe ranked from 79.46 in Austria to 47.62 in Hungary. Spain, with an index of 78.42, was the third country in Europe and the seventh in the world to be ranked in a high position, which is a positive sign for the Spanish NHS.

The Euro Health Consumer Index in 2018 assigned 19th place to Spain among 35 European countries and affirmed that medical excellence can be found in many Spanish places. It recognized that Spain has a large regional variation and the Spanish rely on private care for real excellence. The Spanish Constitution, the main law in the country, establishes the right to health protection and healthcare for all citizens. The Spanish NHS is publicly funded and it is characterized by the universality and gratuity of health services at the time of use; however, there exists, in the country, a parallel private health system, which is optional and complementary to public service.

In 1954, Koos [12] said that patients’ opinions about the healthcare received had to be taken into account and consideration of the patients’ views as a measure for healthcare outcome was advanced by Donabedian [13]. The concept of perceived service quality, however, was first proposed by Gronroos in 1982 [14]. Thus, the idea of identifying quality with the effectiveness of medical treatments was extended to include patient satisfaction as a requirement for good clinical practice. This is how healthcare is considered by the European Foundation Quality Management (EFQM) and the International Organization for Standardization (ISO) [15]. The EFQM model considers that patient satisfaction represents 20% of the total value [16]. Today, patient satisfaction is thought to be the best indicator in the evaluation and improvement of quality in healthcare services [17,18,19]. Indeed, Busse et al. [20] analyzed how the definitions of healthcare quality have evolved from the exclusive consideration of health professionals’ opinion to the consideration of preferences and views of patients.

Patient satisfaction is a useful instrument to value the healthcare process since it provides information about the perceived quality and therefore, can be incorporated in a program of valuation and quality improvement [21,22]. The citizens’ opinions offer the necessary information to adequately manage health resources [23]. For this reason, patient satisfaction has been used to measure the performance of the NHS [24], since it is an excellent indicator of its quality and effectiveness [4]. A high degree of patient satisfaction is usually linked to advanced compliance of the treatments and therefore, it is a signal of health success [1].

Patient satisfaction is a consequence of the interaction between the needs, expectations, and experiences of patients [25]. If the result of healthcare is measured by patient satisfaction with the result achieved, the NHS will be able to satisfy not only the patient’s needs but also their expectations, since satisfaction is understood as the difference between the patient’s expectations and his degree of perception of the public service [26,27,28,29]. When the perceived performance matches or beats the expectations, the service is considered satisfactory. If it does not, the patient is dissatisfied [30]. A previous research affirmed that an unsatisfactory experience influences patient satisfaction more than a good experience [31].

In recent decades, studies about patient satisfaction have been generalized, but their use as a management tool is still unusual [3] because they used to focus more on health personnel (such as physicians and nurses). Without a doubt, a better understanding of how the satisfaction is structured can provide useful knowledge to the managers of health services to implement proper measures, which stimulate the improvement of the services [32]. The decreasing financial resources caused by worldwide crisis and population aging require strategies to improve the service quality in order to maximize patient satisfaction with the lowest possible costs [8,33,34]. Since patient satisfaction allows the evaluation of NHS quality, the development of this type of studies is a measure of responsibility because they provide information on the policies to be implemented and on political programs for healthcare [9,35]. The analysis of patient satisfaction must provide information to healthcare professionals as well as to managers and administrators of the NHS.

Accordingly, the aim of this study was to determine which indicators, among those with available information, positively or negatively influence patient satisfaction in the Spanish NHS, and can provide useful information to system managers. This paper makes the following contributions to the existing literature. First, it provides empirical evidence on the Spanish NHS, which has been scarcely analyzed in this context. Moreover, this paper considers a wide time interval (2005–2016). Second, we used a methodology scarcely used in this sector to date and with this purpose. Third, the study analyzed the influence of expenditures, resources allocation, and safety on patient satisfaction through a wide array of variables, which led us to interesting conclusions.

### Literature Background and Hypotheses

Patient satisfaction is the result of multiple factors, so it is a complex and multidimensional construct. Therefore, its measurement is difficult, and studies about patient satisfaction are heterogeneous, that is, there is a lack of standardization in the methods used to measure patient satisfaction [36,37,38].

There exists an array of questionnaires and scales to measure satisfaction in the service sector. The most widely used scale is SERVQUAL [39,40], developed by Parasuraman et al. [26]. SERVQUAL considers five dimensions of service quality [28,41]: tangibles, assurance, empathy, reliability, and responsiveness. The primary variable that can be controlled by political managers is tangibility or, in other words, resource allocation, which was considered in our study.

Items related to human quality are highly significant to measure patient satisfaction and they have frequently been used in previous studies [42,43]. This kind of variable is not directly controlled by policy makers and therefore, it is not included in public studies, which explains the absence of available public data. In recent years, satisfaction related to a physician’s gender has also been studied [44]. In this respect, we considered that managers should not make gender distinctions, but consider a doctor’s ability regardless of gender.

Since physicians are the most visible element of the healthcare service, it is important to determine patient satisfaction related to family and specialist doctors. Family doctors used to be on the upper levels of satisfaction [45,46] because they are closer to the patients. This indicator leads to greater satisfaction, which, in turn, implies greater compliance and adherence to treatment. This circumstance is the main objective of a healthcare service [4]. Currently, it is not enough that the physician provides competent medical care [47] since patient-centered communication increases satisfaction [48]. For this reason, although patients remember less than half of the information supplied by their doctors [49], the quality and quantity of information received is essential for decision-making by patients [50]. Therefore, the information received in consultation with a specialist doctor is an essential key to patient satisfaction.

The previous literature found a positive relationship between patient satisfaction and healthcare expenditure, in such a way that a higher per capita expenditure in the NHS was associated with higher patient satisfaction [15,51]. In Spain, where health competencies are assigned to the autonomous communities and therefore, budget allocation is uneven across the country, differences in patient satisfaction have been observed [1]. Specifically, the north of the country, where the wealthiest communities reside, showed higher levels of satisfaction than the poor communities located in the south [4].

The most widespread variable in studies on patient satisfaction is resource allocation [3,52]. It generally involves infrastructure [30,32], availability of material and equipment [29,53], tangibility [33], or physical facilities [2]. Medical staff is also included in this concept when used in a broad sense [15]. All the analyzed studies found that a more significant resource provision leads to higher patient satisfaction.

In 2004, the World Health Organization officially authorized the World Alliance for Patient Safety. The intention was to promote patient safety worldwide. This medium reduces the adverse health consequences of unsafe medical care because there are too many safety claims and therefore, much to improve in this area [54]. Security is positively related to quality [30,33]. As a consequence, higher security increases patient satisfaction [2,52,55,56].

The level of use, often referred to as access [2], is generally related to patient satisfaction [3,21,52]. Nevertheless, the direction of the relationship between the two variables is not always the same, since there is a positive association in some cases and negative in others. The main reason for this is the variable of use considered. For example, the longer the length of the hospital stay, the lower the satisfaction [57]. Analog behavior has the number of surgical interventions; however, the outpatient surgery percentage of the number of hospital admissions shows a positive relationship with patient satisfaction [58]. The inverse relationship that links both variables can be explained by considering the satisfaction as the result of the need to continue using services that previously did not meet expectations; that is, it is the result of unsatisfactory service [36].

Gross domestic product (GDP) per capita has been included in some prior studies. In this sense, Europe has been found to have greater patient satisfaction in people with higher incomes [15,59]. Nevertheless, prior research conducted in Spain established that GDP per capita is not related to patient satisfaction [1].

The literature has considered personal variables, such as age, gender, and health condition [60]. The results are not conclusive, especially with respect to age and gender, which have been found not significant in most cases [1,55]. On the contrary, there is consensus with respect to a health condition [53,59], which has been found to be significant, by considering that the most satisfied patients had a greater mortality risk [51]. In our study, life expectancy at birth was the variable analyzed, since we understood that this variable includes, in some way, the three mentioned variables.

Based on the discussed literature, the following hypotheses emerged to provide the scope and depth of this study:

**Hypothesis** **1** **(H1).** *Expenditures positively influence patient satisfaction.*

**Hypothesis** **2** **(H2).** *Expenditures positively influence resource allocation.*

**Hypothesis** **3** **(H3).** *Resource allocation positively influences patient satisfaction.*

**Hypothesis** **4** **(H4).** *Safety positively influences patient satisfaction.*

**Hypothesis** **5** **(H5).** *Resource allocation positively influences the level of use of resources.*

**Hypothesis** **6** **(H6).** *GDP per capita positively influences patient satisfaction.*

**Hypothesis** **7** **(H7).** *Life expectancy at birth positively influences patient satisfaction.*

**Hypothesis** **8** **(H8).** *Resource allocation mediates the relationship between expenditures and patient satisfaction.*

## 2. Materials and Methods

### 2.1. Data and Sample

We conducted a study using data from the perception of Spanish patients. The data of the variables were obtained from the publication made by the Spanish Ministry of Health, Social Services, and Equality (MHSE). This institution has a statistical portal on the means of each autonomous community of the key indicators of the national health system. We used data from 2005 to 2016; hence, the study encompassed 12 years. The data were provided by the information system of the NHS, the National Statistics Institute, and the Spanish Agency of Medicines and Health Products. The maintenance of the indicator set corresponded to the Health Information Institute. The public had no detailed information about the questionnaire, the participation, and so on. Thus, in this study, we only had access to the data, but not to its gathering process.

The European Core Health Indicators (ECHI) model was adapted to the characteristics of the Spanish national health system and its information system and strategic priorities. Other national (Andalusia) and international (Organization for Economic Co-operation and Development, World Health Organization) models were also taken into account, but compatibility with the ECHI indicators was always kept.

Table 2 provides a summary of all the variables and the indicators included in the model, their acronyms, and the data source used. Table 3 shows the mean and standard deviation in 2005 and 2016 for each variable and the variation in the total period.

Previous studies [61,62,63] showed how structural equation modeling (SEM) could become an indispensable tool for managers, policymakers, and regulators in the healthcare sector. Our data came from indexes, as mentioned above, and were contained in a database; therefore, they were secondary data. The primary constructs included in our research model met the requirements of a composite measurement model [64,65].

In this article, we used a partial least square (PLS-SEM) composite scheme to represent the total variance of the variables [66] for different reasons. In particular, PLS-SEM was an adequate model in the case of inclusion of archival information or secondary data [67]. Moreover, PLS composite scheme mode B estimation takes into account the collinearity between the items, giving less weight to the most redundant indicators. Furthermore, as Becker et al [68] suggested, we chose indicators as composite mode B, except patient satisfaction, because the estimation of the weights optimizes *R*^2^ in-sample prediction. We chose composite mode A for patient satisfaction because of collinearity between the indicators [68,69]. All the issues, as mentioned above, summarized the statistical characteristics of the data available in this research.

**Figure 1:**
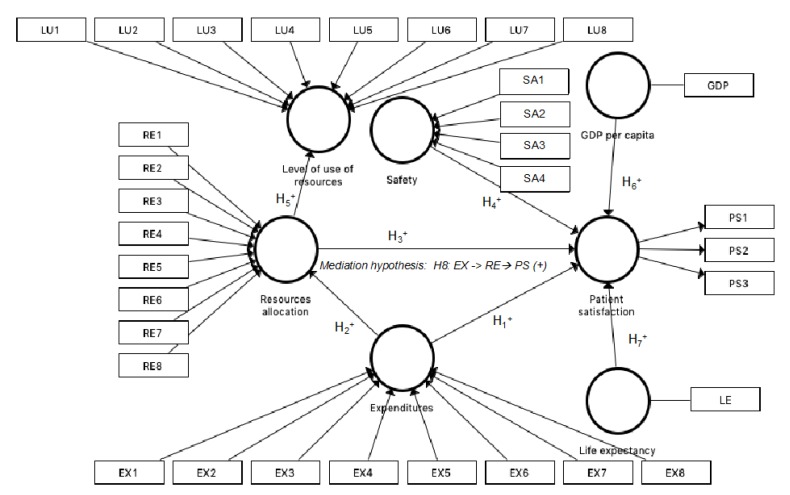
Research model and hypotheses.

Figure 1 depicts the theoretical model proposed in our study. As Figure 1 shows, we examined the relationship between expenditures, resource allocation, and safety over patient satisfaction (Hypotheses 1, 3, and 4). In addition, we analyzed the influences of expenditures on resource allocation (Hypothesis 2), and resource allocation on the level of the use of resources (Hypothesis 5). The influence of the control variables, GDP per capita and life expectancy, was studied on the latent variable, patient satisfaction (Hypotheses 6 and 7). Finally, a mediation effect of resource allocation was analyzed through Hypothesis 8. Expenditures have a direct effect on patient satisfaction, but also an indirect effect through resource allocation. The previous literature has exposed similar effects [70,71]. In our case, we postulated a positive mediation effect and that, at the same time, the simple and direct effects reflected in H2 and H3 are positive. It would be a problem if the hypotheses contradicted each other.

### 2.2. Measurement Variables

The selected variables were based on the secondary data from MHSE, which, in turn, had been based on the conceptual model suggested by ECHI. All the variables associated with each construct are shown in Table 2. In particular, the following constructs were part of our model:

*Patient satisfaction:* The dependent variable was measured by the endogenous variable, patient satisfaction. One of the critical components of quality is the ability of the system to respond to patient preferences, attitudes, and expectations. Patient-centered care is defined as that which establishes an adequate interrelation between professionals and patients to ensure that the decisions made regarding their care process take into account their needs, desires, and preferences. Analogous to the business model of customer satisfaction, patient satisfaction could serve as a patient-centered focus for increasing the care experience in a national health system. There are three indicators of patient satisfaction: (i) degree of satisfaction of the citizen with the information received in the consultation of the specialist doctor about their health problem, (ii) degree of citizen satisfaction with knowledge of the history and monitoring of their health problems by the family doctor and pediatrician, and (iii) degree of satisfaction of citizens with the functioning of the public health system. These indicators were measured by a Likert scale ranging from 1 to 10. Patient satisfaction reflects the patient’s perception of the entire care process and the improvement of satisfaction metrics is within the power of an institution.

*Expenditures:* One of the three exogenous variables was expenditures. It is defined as the disbursement of goods and services intended to preserve, maintain, recover, or improve the health level of a population. When there are limitations in the budget of the healthcare system, the cost-effectiveness analysis can guide policymakers in resource allocation decisions. All the expenditure variables considered refer to the public sector; in other words, they were public budget data.

*Resource allocation:* The second exogenous variable was resource allocation. A high-quality health benefit requires the availability of sufficient resources to meet individual and population needs. The capacity of the system refers to economic resources, infrastructure, equipment, human resources, medical devices, and medicines.

*Safety:* The third exogenous construct refers to the process by which the healthcare system provides safe patient care. It involves minimizing the unnecessary risk of harm to the patient. Healthcare that promotes patient safety in the provision of care implies risk management; declaration, analysis, and monitoring of incidents; and implementation of solutions to minimize incidents.

*Level of use:* This endogenous variable is defined as the use made by citizens of health services. Resource allocation may, in turn, determine the level of use that patients make of such resources.

*Control variables:* Two control variables were studied (GDP per capita and life expectancy at birth) to research their impact on the endogenous variable, patient satisfaction.

### 2.3. Statistical Procedure

The structural equation model was analyzed in a two-step process [72]. We first described the results for the measurement model, which specifies the relationships between constructs and their indicators, before those relating to the structural model, which contains the relationships between constructs or the hypotheses of the model.

(i) Analysis of the measurement model.
(ii) Analysis of the structural model.

This sequence ensured that the measurement scales were valid and reliable before attempting to reach conclusions about the hypotheses included in the structural model [73]. This study applied Smart-PLS 3.2.7 software (SmartPLS GmbH, Bönningstedt, Germany) [74].

## 3. Results

This section details the results obtained for the proposed research model.

### 3.1. Measurement Model

#### 3.1.1. Composite Mode A

The composite measurement model in mode A (patient satisfaction) was assessed in terms of individual item reliability, construct reliability, convergent validity, and discriminant validity.

**Figure 2:**
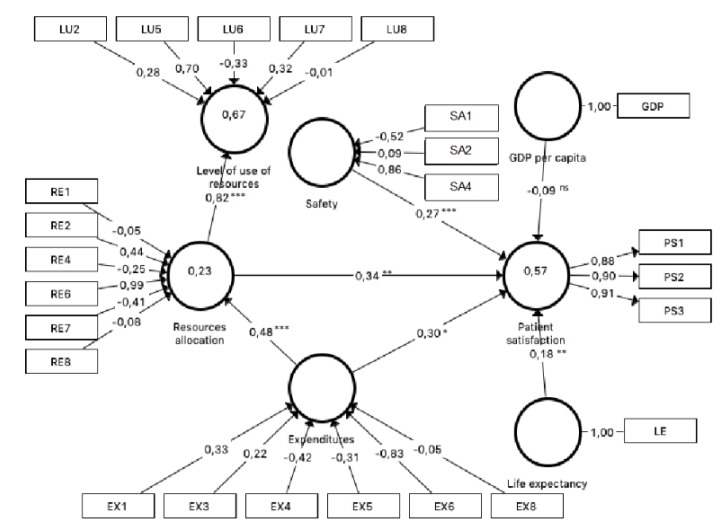
* *p* < 0.05; ** *p* < 0.01; *** *p* < 0.001; ^ns^ not significant. Whole model results.

First, the individual item reliability was analyzed through the loadings. As Figure 2 illustrates, the loadings exceeded the cut-off value of 0.708. Second, Cronbach’s alpha, Dijkstra–Henseler’s rho coefficients, and composite reliability were used to evaluate construct reliability. As Table 4 shows, the construct exceeded the recommended cut-off value of 0.7 for these three measurements. Third, convergent validity was proven since the average variance extracted (AVE) for the construct was higher than 0.5. Table 4 shows that the measurement model was satisfactory concerning the above criteria.

Table 5 presents the results for discriminant validity through the Heterotrait-Monotrait ratio of correlations (HTMT) inference. All the constructs reached discriminant validity because no confidence interval contained the value of zero. This circumstance meant that each variable was different from the others [75].

The data examined above in the measurement model showed that the measures of the construct, patient satisfaction, were reliable and valid.

#### 3.1.2. Composite Mode B

The composite measurement model in mode B was assessed in terms of collinearity among indicators, significance, and the relevance of outer weights.

First, it carried out a process of discarding indicators, which was performed when the indicator exceeded the value of variance impact factor (VIF = 3). As a result of this process, only the indicators shown in Table 2 remained without collinearity.

Second, the relevance of weights was analyzed. Figure 2 shows the relevance of the indicators within their construct. Thus, for the latent variable, expenditures, the items more positively relevant were EX1 (territorialized public health expenditure per protected inhabitant) and EX3 (percentage of health expenditure in primary care). In addition, EX4 (percentage of spending dedicated to concerts), EX5 (percentage of expenditure on intermediate consumption), and EX6 (percentage of pharmacy spending) presented negative relevance.

Concerning resource allocation, the items more positively relevant were RE6 (number of hemodialysis equipment in operation per 100,000 inhabitants) and RE2 (Number of the hospital beds in operation per 1000 inhabitants). On the other hand, the items that presented negative relevance were RE4 (number of posts in day hospitals per 1000 inhabitants) and RE7 (number of hemodynamic equipment in operation per 100,000 inhabitants).

The most positively relevant item for safety was SE4 (reporting rate of adverse drug reactions). Moreover, SE1 (hospital infection rate) showed negative relevance. About the level of use, the items more positively relevant were LU5 (rate of use of hemodialysis per 1000 inhabitants/year), LU7 (rate of use of NMR for 1000 inhabitants/year), and LU2 (outpatient surgery percentage). The item presenting a negative influence was LU6 (rate of use of hemodynamics per 1000 inhabitants/year).

Finally, to assess significance, one can start bootstrapping with 10,000 sub-samples in order to check whether outer weights are significantly different from zero, that is, the recommended minimum by Hair et al. [76]. Since the weights provide information about their contribution, they can be ranked regarding their respective composite [77]. Indicators with a nonsignificant weight, but with significant loadings of 0.50 or higher, were considered relevant [72], which was the case for LU2 and LU7 (Table 6).

### 3.2. Structural Model

Once it was verified that the measurements of the constructs were appropriate, the assessment of the structural model was conducted.

Path coefficients and their 10,000 resampling bootstrap significance levels are reported in Table 7 and Figure 2. In addition, Table 7 shows that constructs’ VIF ranged from 1.000 to 1.700, suggesting that collinearity was not a problem. Moreover, this study assessed quality by checking that the overall predictive relevance of the model by *Q*^2^ value was above zero. It suggested a good fit in model prediction.

Our results suggested that GDP per capita, as a control variable, had no significant impact on patient satisfaction, so H6 was rejected. Expenditures, resource allocation, and safety had a positive and significant impact on patient satisfaction (*p* = 0.020, *p* = 0.002, *p* = 0.000, respectively), as well as life expectancy at birth *(p* = 0.017)*;* hence, H1, H3, H4, and H7 were supported. Furthermore, the direct effects between expenditures to resource allocation and resource allocation to the level of use had a positive and significant impact (*p* = 0.000, *p* = 0.000, respectively); therefore, H2 and H5 were supported as well.

These results followed the mediation effect since the total effect of expenses on patient satisfaction can be addressed by adding direct and indirect effects. The mediation hypothesis (H8) was analyzed when the indirect effects were significant [78]. The indirect effect of expenditures on patient satisfaction through resource allocation was positive and significant (*p* = 0.007), supporting H8 (Table 7). Moreover, the direct effect was also significant, which indicated that the mediation effect was partial [79]; that is, expenses influenced patient satisfaction directly (H1), but also indirectly through resource allocation. The value of Variance Accounted For (VAF) indicated that the mediated proportion was 35.2% of the total effect of expenditures on patient satisfaction (see the indirect effect in Table 7).

The determination coefficient (*R*^2^) exceeded 0.1 for the endogenous latent variable [80], so the constructs had an acceptable quality of prediction power.

## 4. Discussion

We analyzed 33 variables to identify indicators capable of influencing patient satisfaction in the Spanish context. To do that, partial least squares (PLS-SEM) was applied to data from 2005 to 2016. The influence of expenditures, resource allocation, and safety constructs on patient satisfaction was tested. Resource allocation showed a positive influence on the level of use construct. The number of hospital beds, hemodialysis equipment, rate of adverse drug reactions, and expenditures positively influenced patient satisfaction. In contrast, the number of posts in day hospitals, the hospital infection rate, and the percentage of pharmacy spending negatively influenced patient satisfaction. The control variable, life expectancy, positively influenced patient satisfaction, but GDP was not significant.

It is necessary to integrate patients’ opinions into the management of NHS, and studies about satisfaction enable this to be done. In this sense, this work was carried out to provide useful information to NHS managers about some variables and their influence on patient satisfaction. This would allow the implementation of health policies to improve the perception of the provision of services by their users.

It is important to note that to date, the specialized literature has offered results aimed primarily at healthcare professionals (physicians and nursing staff, fundamentally), but studies aimed at administrators and managers of NHS are scarce. For this reason, the variables of this study did not refer to a specific patient (age, gender, etc.) and their relationship with professionals (politeness given by healthcare professionals, patient participation in decision-making, etc.). On the one hand, we used variables of investments and results of health practice. On the other hand, we determined an increase in the level of satisfaction by studying many variables simultaneously.

We wanted to indicate that patient satisfaction with both family doctors and specialist doctors was higher than patient satisfaction with the functioning of the NHS. Thus, physicians were located in the upper echelons of assessment, in line with previous studies [45,46].

This research analyzed the influence of expenditures, resource allocation, and safety on patient satisfaction, as well as the resource allocation on the level of use of the Spanish NHS. The aim was to increase knowledge for managers and the government on how these three latent variables were perceived by patients to value their satisfaction. Moreover, we introduced two control variables: GDP per capita and life expectancy. The proposed model confirmed seven of the eight causal relationships established, which explained 57.1% of patient satisfaction. The two most influential variables were resource allocation and expenditures, with similar weights, followed by safety and, finally, life expectancy.

A positive effect of resource allocation on patient satisfaction was found, in line with previous studies, which established that resources, such as facilities, explain a critical part of satisfaction [15,30,32] widely. We observed that the number of hospital beds in operation had a decisive weight in the construct, while the number of posts in day hospitals had a negative influence. This fact implied that citizens preferred that their health problems be solved as in-patients instead of outpatients. Previous research found that satisfaction is higher in in-patients [51,59]. The trend in Spain is a shift from more expensive in-patient care toward outpatient care [81]. Therefore, this change should be sufficiently explained to patients with the aim of showing them the advantages of such care. The number of hemodialysis equipment was an important variable that influenced the latent variable. This equipment was vital for the survival of a part of the population, and we observed a significant difference between autonomous communities. In general, the wealthiest communities have a better provision of equipment, which explained the mediation effect of resource allocation between expenditures and patient satisfaction, since most impoverished communities showed the lowest satisfaction with the NHS [1,4,51].

Expenditures were also found to exert a positive influence on patient satisfaction, but with a slightly lower weight than resource allocation. This relationship was in agreement with prior literature [51]. The territorialized public health expenditure per protected inhabitant exerted a direct influence on satisfaction, which is in line with the results reported by Pérez-Romero et al. [15]. The percentage of pharmacy spending presented an inverse relation with patient satisfaction; Valls and Abad [58] found a similar relationship. In this sense, it is interesting to highlight that higher expenses in drugs influence satisfaction positively only in patients over 65 years old [36], which explained why drug expense negatively influenced patient satisfaction.

Safety directly affected patient satisfaction, but to a lesser extent than the two constructs previously analyzed, which is in line with previous research [2,52,56]. Thus, more safety implied higher satisfaction, which implied that hospital safety was perceived as a fundamental indicator of patient satisfaction. We observed that the hospital infection rate negatively affected patient satisfaction, which is rational, and it is in concordance with the results of Valls and Abad [58]. If a higher rate of adverse drug reactions positively affects patient satisfaction, we must assume that the treatment and attention received by patients is adequate and, consequently, that they perceive technical competence as satisfactory. They feel that the NHS is functioning properly [36]. In fact, since 2005, the Spanish authorities have established measures intended to improve patient safety, such as awareness of medical staff and patients or safety research [81].

The results showed that resources and the level of use were positively related. We assumed that the influence of resource allocation on the level of use could be related to the demands of sanitary equipment according to the diseases of the citizens, although the goal of the NHS is to improve the service by reducing costs [8,34]. In Spain, some complex diagnostic and treatment procedures are limited, and patients have to suffer long waiting lists. In areas where resources are more considerable, patients have better care, such as, more hemodialysis and CT equipment available for use. We mentioned the important variations across regions in Spain. On the other hand, and after considering the overuse of many surgical procedures, the “Commitment for the Quality of the Scientific Societies in Spain” aims to reduce unnecessary surgical interventions through an array of “do not do” recommendations about specific health services [81].

Regarding control variables, we found that GDP per capita did not exert any influence on patient satisfaction, which is in line with recent research performed in the Spanish context [1]. In this vein, the literature is not conclusive since other authors found a positive influence [15]. On the contrary, life expectancy at birth directly affected patient satisfaction and therefore, patients with more life expectancy valued the NHS more positively. In Spain, the NHS is different across the country, since health competencies have been transferred to autonomous communities and, accordingly, the quality of NHS is not homogeneous in all the territories. Hence, health population and life expectancy were influenced [8,30], which impact patient satisfaction. It was interesting to observe (Table 3) that patient satisfaction had increased in the period of 2005–2016, especially satisfaction with family doctors and pediatricians. Expenditures increased more than GDP. It is remarkable that expenditures for training residents experienced the greatest growth, while the percentage of pharmacy spending decreased. It is noticeable that Spain is containing drug spending through the entry of generics in the market; in fact, pharmacists must substitute the medicine prescribed by the cheapest generic. Regarding resources, we noticed that the increase was, in general, below the level of expenditures. In fact, health expenditure per capita in Spain was below the European Union average; for example, in 2015, Spain accounted for 9.2% of GDP, while the EU was 9.9% [81]. Finally, if we observe the level of use, we can highlight the increase in outpatient surgery percentage to above 22%.

The research in this article provides outstanding data on patient satisfaction with the Spanish NHS. Relevant factors that affect the perception of public health by citizens were identified. These factors are the number of hospital beds in operation, positions in day hospitals, hemodialysis equipment, percentage of outpatient surgery, CT use rate, NMR use rate, adverse drug reaction rate, hospital infection rate, public health spending, and percentage of pharmacy expenditures.

The results derived from this research give useful evidence to NHS managers and they provide valid information to help in the design and implementation of health policies, which lead to an improvement in the quality of provided services and, therefore, to greater patient satisfaction, since the analyzed variables explained 57.1% of patient satisfaction.

The main limitation we found in this study was the availability of information. It would have been interesting to know the selection process of interviewed population in order to establish generalizations. Moreover, it would be convenient to have data on other variables, which could be considered in the analyses of patient satisfaction, such as social variables (education level, poverty rate, etc.), success rate of treatments received, and sex disaggregated data. We understand that the method would be more efficient if we had had access to all the patients’ surveys instead of the mean values of the surveys, but unfortunately, such data are not at public disposal. Similar research is needed to analyze this subject in other national and or regional healthcare services and with other secondary data.

We consider that it is necessary to deepen this kind of study in the future since, so far, most of the research has been focused on localized studies (for example, in a particular hospital) and preferably oriented to health personnel (mainly, physicians and nurses). The lack of available data at the national level has led to a shortage of research based on patient satisfaction and aimed at the design, application, and valuation of global health policies.

## 5. Conclusions

Understanding the influence of the complex interaction between expenditures, resource allocation, and safety to patient satisfaction allows informed decision-making to improve public health in the Spanish system. Using the structural equation modeling approach, we developed a patient satisfaction model. It allows for an impact comparison of the antecedent variables and provides the public agencies and policymakers critical information on variables with which they can make informed decisions. In this vein, according to our results, the number of hospital beds in operation and hemodialysis equipment were proven to be positively associated with patient satisfaction in our model. Likewise, the posts in day hospitals showed a negative influence. In this sense, we suggest that more attention is needed on patient satisfaction, while promoting the shift from in-patient to day care settings. Moreover, hospital infections must be controlled and minimized. Regarding public health expenditure, greater effort in this sense indicated increased patient satisfaction.

## Figures and Tables

**Table 1:** Healthcare index by country.

Region	Analyzed Countries	Range
World	89	86.69–39.35
Africa	7	63.27–40.17
America	15	71.27–40.17
Asia	30	86.89–41.32]
Europe	35	79.46–47.62
Oceania	2	76.82–73.71

**Table 2:** Data description and source.

Composites	Indicators	Description
Patient satisfaction (mode A)	PS1	Degree of satisfaction of citizens with the functioning of the public health system.
	PS2	Degree of citizen satisfaction with knowledge of the history and monitoring of their health problems by the family doctor and pediatrician.
	PS3	Degree of satisfaction of the citizen with the information received in consultation with a specialist doctor about his health problem.
Expenditures (mode B)	EX1	Territorialized public health expenditure, per protected inhabitant.
	EX2 *	Percentage of the cost of specialized care services.
	EX3	Percentage of health expenditure in primary care.
	EX4	Percentage of spending dedicated to concerts.
	EX5	Percentage of expenditure on intermediate consumption.
	EX6	Percentage of pharmacy spending.
	EX7 *	Percentage of staff compensation expense.
	EX8	Percentage of health expenditure destined to training residents.
Resources (mode B)	RE1	Number of medical staff in specialized care for 1000 inhabitants.
	RE2	Number of hospital beds in operation per 1000 inhabitants.
	RE3 *	Number of operating theaters for 100,000 inhabitants.
	RE4	Number of posts in day hospitals per 1000 inhabitants.
	RE5 *	Number of computerized axial tomography (CT) equipment in operation per 100,000 inhabitants.
	RE6	Number of hemodialysis equipment in operation per 100,000 inhabitants.
	RE7	Number of hemodynamic equipment in operation per 100,000 inhabitants.
	RE8	Number of nuclear magnetic resonance (NMR) equipment per 100,000 inhabitants.
Level of use (mode B)	LU1 *	Frequency of hospital admissions per 1000 inhabitants/year.
	LU2	Outpatient surgery percentage.
	LU3 *	Surgical interventions rate per 1000 inhabitants/year.
	LU4 *	Rate of use of CT per 1000 inhabitants/year.
	LU5	Rate of use of hemodialysis per 1000 inhabitants/year.
	LU6	Rate of use of hemodynamics per 1000 inhabitants/year.
	LU7	Rate of use of NMR for 1000 inhabitants/year.
	LU8	Frequency in specialized care consultations per 1000 inhabitants/year.
Safety (mode B)	SA1	Hospital infection rate.
	SA2	Overall in-hospital mortality per 100 hospital discharges.
	SA3 *	In-hospital mortality after surgical intervention per 100 surgical discharges.
	SA4	Reporting rate of adverse drug reactions.
GDP per capita (control variable)	GDP	Gross domestic product per capita.
Life expectancy (control variable)	LE	Life expectancy at birth.

Source: Ministry of Health, Social Services, and Equality (MHSE), 2005–2016. * These indicators were not included in latent variables due to the multicollinearity criteria of PLS-SEM.

**Table 3:** Mean, standard deviation, and variation in the period 2005–2016.

Composites	Indicators	Mean 2005	Standard Deviation 2005	Mean 2016	Standard Deviation 2016	Variation 2005–2016
Patient satisfaction	PS1	6.25	0.45	6.73	0.38	7.68
	PS2	7.14	0.37	7.72	0.25	8.12
	PS3	7.06	0.48	7.31	0.35	3.54
Expenditures	EX1	1157.56	80.89	1462.79	131.19	26.37
	EX2	56.66	3.17	63.55	3.33	12.16
	EX3	14.31	2.01	14.07	1.74	−1.68
	EX4	7.53	7.51	7.38	5.09	−1.99
	EX5	19.22	2.36	25.95	3.23	35.02
	EX6	22.97	2.77	16.83	1.99	−26.73
	EX7	44.72	5.96	47.74	4.42	6.75
	EX8	1.20	0.36	1.70	0.40	41.67
Resources	RE1	90.78	6.58	92.22	6.10	1.59
	RE2	73.94	10.84	79.47	9.85	7.48
	RE3	67.88	8.98	73.01	9.41	7.56
	RE4	78.90	19.92	87.06	10.84	10.34
	RE5	67.90	12.03	73.03	11.24	7.56
	RE6	84.21	15.51	90.44	11.39	7.40
	RE7	61.58	22.87	66.11	16.45	7.36
	RE8	46.60	16.35	56.05	13.43	20.28
Level of use	LU1	76.61	11.34	79.31	10.98	3.52
	LU2	35.19	7.54	43.07	6.24	22.39
	LU3	70.01	10.86	72.76	10.87	3.93
	LU4	83.68	10.69	85.29	9.80	1.92
	LU5	78.67	25.07	89.23	14.61	13.42
	LU6	74.73	24.94	86.61	12.61	15.90
	LU7	15.43	3.15	39.11	15.67	153.47
	LU8	1459.08	232.05	1722.69	242.03	18.07
Safety	SA1	1.19	0.32	1.05	0.26	−11.76
	SA2	4.13	0.46	4.78	0.61	15.74
	SA3	1.77	0.30	1.72	0.25	−2.82
	SA4	243.53	174.71	588.37	418.93	141.60
GDP per capita	GDP	20.99	4.01	23.50	4.96	11.96
Life expectancy	LE	80.45	0.84	83.49	0.78	3.78

**Table 4:** Measurement validation.

Composite ^1^	Cronbach’s Alpha	Dijkstra–Henseler’s Rho	Composite Reliability (CR)	Average Variance Extracted (AVE)
Patient satisfaction	0.877	0.878	0.924	0.803

^1^ Patient satisfaction was measured as a mode A composite.

**Table 5:** HTMT inference.

HTMT Inference *	Original Sample	Sample Mean	5.0%	95.0%
Life expectancy → GDP per capita	0.478	0.477	0.388	0.558
Patient satisfaction → GDP per capita	0.152	0.170	0.119	0.243
Patient satisfaction → Life expectancy	0.500	0.499	0.394	0.598

* Significance, 95% bias-corrected confidence interval performed by a bootstrapping procedure with 10,000 replications.

**Table 6:** Significance of weights.

	Original Sample	*t*	Loadings	Lo95	Hi95
Expenditures					
EX1	0.332 *	1.910	0.673	[0.006;	0.578]
EX3	0.221 ^ns^	1.775	0.368	−0.018	0.395
EX4	−0.419 ^ns^	1.642	−0.261	−0.652	−0.113
EX5	−0.309 ^ns^	1.424	0.049	−0.523	0.024
EX6	−0.834 *	1.971	−0.733	−1.082	−0.350
EX8	−0.051 ^ns^	0.308	0.213	−0.287	0.261
Resource Allocation					
RE1	−0.054 ^ns^	0.397	0.344	−0.283	0.164
RE2	0.440 **	2.421	0.390	0.119	0.584
RE4	−0.248 *	2.030	0.156	−0.368	−0.055
RE6	0.990 **	2.725	0.871	0.661	1.116
RE7	−0.412 ^ns^	1.571	−0.143	−0.664	−0.051
RE8	−0.075 ^ns^	0.342	0.463	−0.423	0.294
Safety					
SA1	−0.520 **	2.907	−0.461	−0.681	−0.304
SA2	0.092 ^ns^	0.669	0.158	−0.133	0.316
SA4	0.865 ***	4.239	0.863	0.713	0.969
Level of use					
LU2	0.276 *	2.183	0.593	−0,024	0.390
LU5	0.695 *	2.263	0.869	0,355	0.948
LU6	−0.329 ^ns^	1.261	−0.161	−0.639	0.058
LU7	0.323 *	1.710	0.564	−0.062	0.555
LU8	−0.007 ^ns^	0.052	0.348	−0.259	0.197

* *p* < 0.05; ** *p* < 0.01; *** *p* < 0.001; ^ns^ not significant. Significance, *t* statistic, and 95% bias-corrected confidence interval performed by a bootstrapping procedure with 10,000 replications.

**Table 7:** Whole sample results.

	Path	*t*	*p*	Lo95	Hi95	*f* ^2^	VIF
Direct effects							
EX → PS	0.303 *	2.055	0.020	0.157	0.40	0.128	1.676
RE → PS	0.338 **	2.947	0.002	0.125	0.423	0.198	1.342
SA → PS	0.272 ***	3.370	0.000	0.184	0.355	0.151	1.146
GDP → PS	−0.086 ^ns^	0.956	0.170	−0.246	0.051	0.013	1.347
LE → PS	0.180 *	2.112	0.017	0.051	0.330	0.044	1.700
*R^2^: 0.57; Q^2^: 0.426*							
EX → RE	0.475 ***	3.734	0.000	0.349	0.672	0.292	1.000
*R^2^: 0.23*							
RE → LU	0.817 ***	21.662	0.000	0.768	0.892	2.003	1.000
*R^2^: 0.67*							
Indirect effect						VAF	
EX → RE → PS	0.161 **	2.450	0.007	0.065	0.223	35.2%	na

* *p* < 0.05; ** *p* < 0.01; *** *p* < 0.001; ^ns^ not significant. Significance, *t* statistic, and 95% bias-corrected confidence interval performed by a bootstrapping procedure with 10,000 replications. VIF: Inner model variance inflation factor; VAF: variance accounted for.
